# LILRA3 Binds Both Classical and Non-Classical HLA Class I Molecules but with Reduced Affinities Compared to LILRB1/LILRB2: Structural Evidence

**DOI:** 10.1371/journal.pone.0019245

**Published:** 2011-04-29

**Authors:** Myongchol Ryu, Yong Chen, Jianxun Qi, Jun Liu, Zheng Fan, Gol Nam, Yi Shi, Hao Cheng, George F. Gao

**Affiliations:** 1 CAS Key Laboratory of Pathogenic Microbiology and Immunology, Institute of Microbiology, Chinese Academy of Sciences, Beijing, China; 2 Graduate University, Chinese Academy of Sciences, Beijing, China; 3 Institute of Microbiology, State Academy of Sciences, Pyongyang, Democratic People's Republic of Korea (DPRKorea); 4 Core Facility, Institute of Microbiology, Chinese Academy of Sciences, Beijing, China; 5 Beijing Institutes of Life Science, Chinese Academy of Sciences, Beijing, China; University Paris Sud, France

## Abstract

Structurally, Group 1 LILR (Leukocyte Immunogloblin (Ig)-Like Receptor, also known as Ig-like transcripts, ILT; Leukocyte Ig-like receptor, LIR; and CD85) members are very similar in terms of the HLAIs (human leukocyte antigen class I molecules) binding region and were hypothesized that they all bind to HLAIs. As one of the Group 1 LILRs, LILRA3 is the only secretory LILR and may greatly control the inhibitory immune response induced by LILRB1, LILRB2, and other HLA-binding LILR molecules like LILRA1. Nevertheless, little was known about the binding of LILRA3 to HLAIs. In this report, we present the crystal structure of the LILRA3 domain 1 (D1) and evaluate the D1 and D1D2 (domain 1 and domain 2) binding to classical and non-classical HLAIs using BIAcore® surface plasmon resonance analysis (SPR). We found that LILRA3 binds both classical HLA-A*0201 and non-classical HLA-G1 but with reduced affinities compared to either LILRB1 or LILRB2. The polymorphic amino acids and the LILRA3 D1 structure support this notion.

## Introduction

Immune cells express activating and inhibitory receptors on their surfaces to allow an adequate immune response. The corresponding ligands binding to activating or inhibitory receptors induce and modulate innate and adaptive immunity [1], [2]. Human chromosome 19q13.4 contains important immune cell receptor genes [3], such as killer cell inhibitory receptors (KIRs), leukocyte immunoglobulin (Ig)-like receptors (LILRs), leukocyte associated Ig-like receptors (LAIRs) and the Fcα receptor (FcαR).

LILRs [4], [5], [6] are also called Ig-like transcript (ILT) or leukocyte Ig-like receptor (LIR) or CD85 and are closely related to KIRs but expressed on the surface of a more broad range of cells (i.e., on lymphoid and/or myeloid cells). There are 13 members (two of them are believed to be pseudogenes) of either activating or inhibitory receptors in the LILR family [7]. LILR family members contain either two or four C2-type Ig-like domains in their extracellular portions (D1, D2, D3, and D4). With the exception of LILRA3, activating receptors (LILRA1–A6) contain a short cytoplasmic tail after the transmembrane domain. They lack any signaling motif but recruit the γ-chain of FcεRI through a charged arginine residue in the transmembrane domain and deliver an activating signal through the γ-chain cytoplasmic immunoreceptor tyrosine-based activating motif (ITAM). In contrast, inhibitory receptors (LILRB1–B5) have a long cytoplasmic tail containing immunoreceptor tyrosine-based inhibitory motif (ITIM) and transduce an inhibitory signal. LILRA3 is the only secreted soluble LILR lacking the transmembrane and cytoplasmic domains [8], [9].

It was suggested [10] that LILRs can be categorized into 2 groups according to the amino acid sequence similarity of the region responsible for binding to human leukocyte antigen (HLA). As opposed to Group 2 LILRs, Group 1 members display high sequence similarity and are predicted to bind to HLA class I molecules (HLAIs) [11], [12], [13]. Among the 13 LILRs, LILRB1, B2, A1, A2, and A3 belong to Group 1. It is well known that LILRB1 and LILRB2 bind to a broad range of classical and non-classical HLAIs [13], [14], [15] and major histocompatibility complex (MHC) class I-like molecules, such as UL18 [16]. Furthermore, LILRA1 binding to HLA-B27 has been reported [17], but a LILRA2 domain-swapped dimer structure [18] is unable to bind any HLAIs. However, among the Group 1 members, only LILRA3 has not been examined in detail concerning HLAI binding.

**Figure 1:**
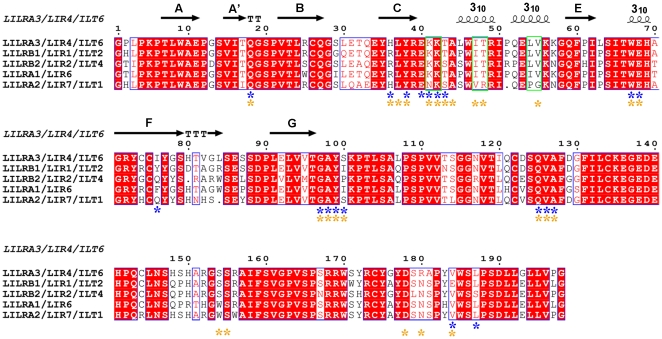
Multiple amino acid sequence alignment of the D1 and D2 domains of Group 1 LILRs. The secondary structure elements of LILRA3 D1 are shown above the sequences. The asterisks indicate the residues involved in the HLAIs binding of LILRB1 and LILRB2 (blue for LILRB1 and yellow for LILRB2). The residues previously hypothesized to be critical to form the 3_10_ helices region in domain 1 of Group 1 LILRs are demarcated with green.

It was previously proposed [18], [19] that the crucial residues that govern binding of Group 1 LILRs to HLAIs are Lys42 and Lys43 (KK), Ile47 and Thr48 (IT), and Leu54 and Val55 (LV) in the D1 domain, and that the hydrophobic 3_10_ helices formed by these residues appropriately orient the key residues for HLA binding. Both LILRB1 and LILRB2 of Group 1 LILRs have these crucial residues in the D1 domain and truly bind to at least 3 alleles of classical and non-classical HLAIs [20]. Meanwhile, LILRA2 [18] as Group 1 LILRs, and LILRB4 [21] and LILRA5 [22] as Group 2 LILRs have no these residues in the D1 domain. Indeed, there is no so called “hydrophobic core” by strands-helices transition in the D1 domain of these LILRs. In contrast, LILRA1 and LILRA3 have the above-mentioned conditions for binding to HLAIs (Fig. 1). However, the structure and evaluation of LILRA3 binding to HLAIs are required to confirm this hypothesis. Further, LILRA3 is the only non-membrane-bound LILR, and its function was predicted to be antagonistic with HLA-binding Group 1 LILRs [23]. Recently, LILRA3 is implicated in the possible pathogenesis of Psoriasis [24], multiple sclerosis [25], Sjogren's syndrome [26] and rheumatoid arthritis [27], indicating its potential significant functions in autoimmune diseases. Thus, from both the structural and functional points of view, it would be interesting to determine if LILRA3 interacts with HLAIs. Though two domains (D1 and D2) take part in the interaction between LILR and HLAIs, previous studies [28], [29] and the co-crystal structures of HLA-A*0201 with LILRB1 [10] and HLA-G1 with LILRB2 [30] indicate that the main contacts (nearly 75% of them) are contributed by the first domain, D1.

Here, we report the binding properties of the LILRA3 D1 and D1D2 domains to HLAIs (classical HLA-A*0201 and non-classical HLA-G1) as analyzed by BIAcore® surface plasmon resonance (SPR) analysis, as well as the crystal structure of the LILRA3 D1 domain. We found that LILRA3 binds both classical and non-classical HLAIs as predicted, but with reduced affinities relative to LILRB1/B2. Further, the LILRA3 D1 crystal structure clearly displays conformational changes in the residues responsible for the binding of Group 1 LILRs to HLAIs, which greatly affects the binding affinity to HLAIs.

## Results

### Soluble protein characterization and BIAcore® binding

**Figure 2:**
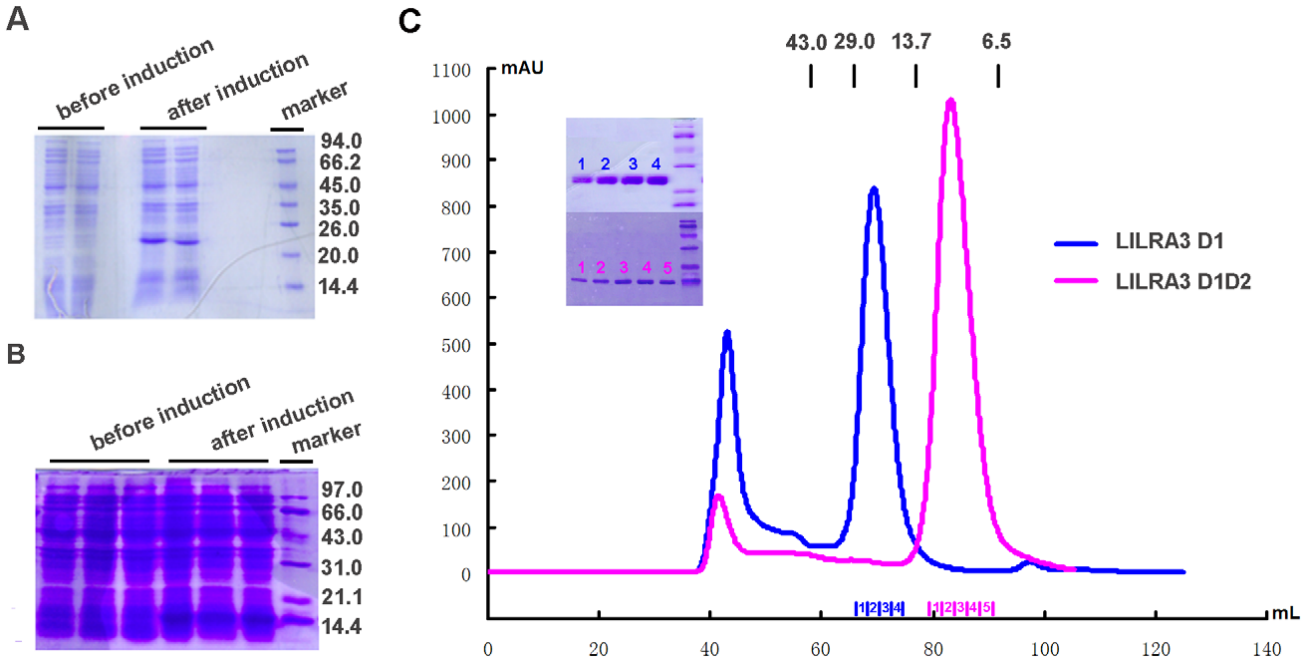
SDS-PAGE analysis of the inclusion body preparations and size exclusion chromatography profiles of LILRA3 D1 and D1D2. A. Induction of LILRA3 D1D2 by ITPG. The first two lanes are the samples before IPTG induction, and the next two are the samples after induction. The last lane is the molecular weight standard (shown in kDa). B. Induction of LILRA3 D1 by ITPG. The first three lanes are the samples before induction, and the next three are the samples after IPTG induction. The last lane is the molecular weight standard (shown in kDa). C. Size exclusion chromatogram showing LILRA3 D1 and D1D2 elution profiles by Superdex 75 10/300 GL column (GE Healthcare). The profiles are marked with the approximate positions of molecular mass standards of 43.0, 29.0, and 13.7 kDa. The size exclusion chromatogram and 12% SDS-PAGE separation profile (inset) of LILRA3 D1 and D1D2 fractions indicated that pure proteins were obtained as monomers.

LILRA3 is a soluble protein, the only secreted member of the LILR family, and contains four Ig-like domains (D1, D2, D3, and D4). The full-length sequence encoding LILRA3 was cloned from a human lymphocyte cDNA library (Strata Gene), and the fragments encoding the LILRA3 D1 or D1D2 regions were cloned from the full-length LILRA3 gene. Based on the previous observation that the first two domains (D1 and D2) of Group 1 LILRs are responsible for binding to HLAIs and to determine if LILRA3 interacts with HLAIs, the first or first and second N-terminal domains of LILRA3 (D1, residues 1–97; and D1D2, residues 1–197, respectively) were expressed in *Escherichia coli* (Fig. 2A and 2B), followed by purification from inclusion bodies, and renaturation via dilution refolding method. Size-exclusion of the refolded materials and SDS-PAGE analysis (Fig. 2C) indicated the correct molecular weight of the single and double Ig-domains [31]. Similarly, far UV circular dichroism (CD) spectra indicated that each sample was mainly comprised of β-sheet secondary structure (data not shown). Thus, the two proteins, LILRA3 D1 and D1D2, were successfully refolded, purified with high purity, and used to evaluate the binding of LILRA3 to HLAIs.

**Figure 3:**
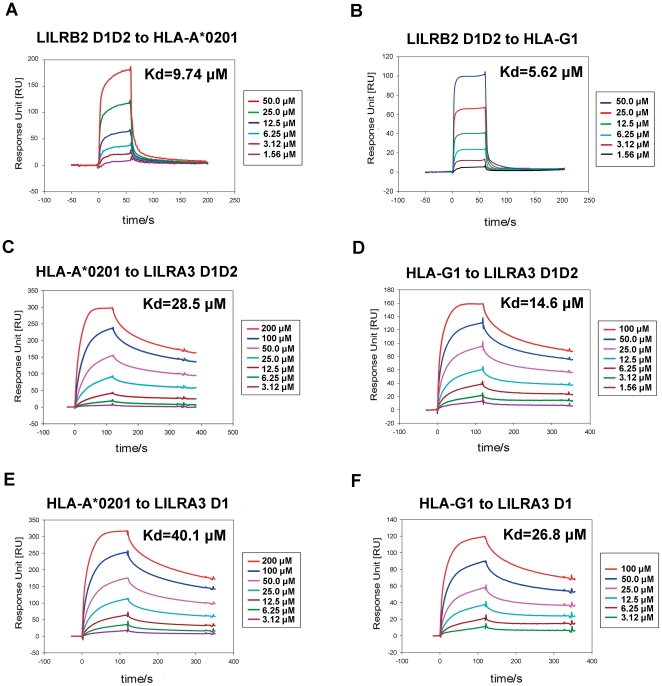
BIAcore® surface plasmon resonance analysis of LILRBA3 D1 and D1D2 monomer binding to immobilized HLA-A*0201 and HLA-G1. A. Kinetic analysis of the LILRB2 D1D2 monomer binding to the immobilized HLA-A*0201 (as a positive control). Protein at the indicated concentrations was injected through the HLA-A*0201-immobillzed flow cells (700 response units (RU)). B. Kinetic analysis of the LILRB2 D1D2 monomer binding to the immobilized HLA-G1 (as a positive control). Protein at the indicated concentrations was injected through the HLA-G1-immobilized flow cells (300 RU). C. Kinetic analysis of the LILRA3 D1D2 monomer binding to immobilized HLA-A*0201. D. Kinetic analysis of the LILRA3 D1D2 monomer binding to immobilized HLA-G1. E. Kinetic analysis of the LILRA3 D1 monomer binding to immobilized HLA-A*0201. F. Kinetic analysis of the LILRA3 D1 monomer binding to immobilized HLA-G1.

We used SPR to compare LILRA3 D1 and D1D2 with LILRB2 for the binding to HLA-A*0201 and HLA-G1. It was previously reported [20], [32] that LILRB2 binds a broad range of classical HLAIs (HLA-A*1101, -B*3501, -C*0401, and -Cw*0702) with affinities (*K*_d_) ranging from ∼14 to ∼45 µM and also binds a non-classical HLA (HLA-G1) with a higher affinity (*K*_d_≈5 µM). First, we evaluated the binding affinity of LILRB2 to HLA-A*0201 and HLA-G1. LILRB2 D1D2 was injected over the CM5 sensor surface on which HLAIs were immobilized using standard amine coupling chemistry. As shown in Figure 3A and 3B, the affinity constants of LILRB2 D1D2 binding to HLA-A*0201 and HLA-G1 were 9.74 and 5.62 µM, respectively, the latter being similar to the previously reported HLA-G1 value [20], [32]. Using the same conditions, LILRA3 D1 and D1D2 were injected over the CM5 sensor surface on which HLAIs were immobilized, and the results are shown in Figure 3C–3F. Both LILRA3 D1 and D1D2 bound to HLA-A*0201 and HLA-G1 with affinities in the range of 20–40 µM. Previous research evaluating the direct binding of LILRs to HLAIs [20] demonstrates that Group 1 LILRs bind with a higher affinity to non-classical than classical HLAIs. Our experiment reconfirmed this trend. For HLA-G1, LILRA3 D1D2 showed a reduced binding affinity (*K*_d_ = 14.6 µM) than LILRB2 D1D2 (*K*_d_ = 5.62 µM), and for HLA-A*0201, it showed a further reduced binding affinity (*K*_d_ = 28.5 µM) similar to that of LILRA3 D1 binding to HLA-G1 (*K*_d_ = 26.8 µM). Meanwhile, LILRA3 D1 displayed much lower binding affinity to HLA-A*0201 (*K*_d_ = 40.1 µM) but still reached the binding affinity range of CD8 (∼100 µM).

To rationalize the structural basis for the binding of LILRA3 to HLAIs, we attempted to characterize the structure of LILRA3 in the HLA-binding region. However, despite using identical refolding and purification procedures, all efforts failed to yield any diffractive crystals of LILRA3 D1D2, and thus, only LILRA3 D1 could be crystallized.

### Crystal structure of the LILRA3 D1 domain

**Figure 4:**
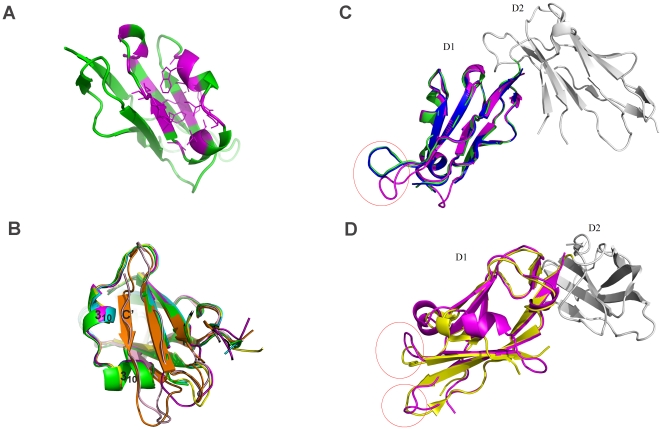
Hydrophobic pocket of LILRA3 D1 and comparison of the crystal structures of LILRA3 D1, LILRB1 D1, and LILRB2 D1. A. The hydrophobic pocket of LILRA3 D1. The hydrophobic residues located around this area are colored with magenta. B. The helix-strand transition between LILRB1/LILRB2/LILRA3 and LILRA2/LILRA5: magenta, LILRA3; cyan, 1GOX (LILRB1); green, 1UFU (LILRB1); yellow, LILRB2 in complex with HLA-G1 (2DYP); orange, 2D3V (LILRA5); and light pink, LILRA2 C. The difference in the loop region of the LILRA3 D1 (pink) superposed on LILRB1 D1D2 (1GOX-green and 1UFU-blue). D. The difference of the loop region of LILRA3 D1 (pink) superposed on the LILRB2 D1D2 (2GW5-yellow).

The overall structure of the LILRA3 D1 domain has characteristics consistent with Ig-like domain structures and also displays properties unique to the LILR family (Fig. 4A). The structure is primarily composed of β strands arranged into two anti-parallel β sheets, with one β sheet containing three anti-parallel β strands (A, B and E) and the other containing four anti-parallel β strands (C, F, G and A′) [18]. Like the crystal structure of LILRB1 D1D2 [28] and B2 D1D2 [33], the D1 structure of LILRA3 also has a pocket-type hydrophobic core surrounded by the C and E strands and two 3_10_ helices between the two strands (Fig. 4A).

Most of the hydrophobic residues on the strands and the 3_10_ helices face toward the interior of this hydrophobic core, which causes the hydrophilic residues on the adjacent loop region to protrude outside of the core. This hydrophobic core not only contains some important residues for direct binding to HLAIs but also pushes the adjacent hydrophilic residues (e.g., Tyr38, Glu40, Lys41, Lys42 and Glu68) to assume a proper conformation for its binding to HLAIs, unlike the residues of Group 2 members and LILRA2, where the two 3_10_ helices between the C and E strands cannot be seen by helix-strand transitions (Fig. 4B). Thus, via the crystal structure of LILRA3 D1, we demonstrated that LILRA3 D1, like LILRB1 and B2, contains a hydrophobic core (as previously predicted [18]) and is able to interact with HLAIs. There is also a disulfide bond between cysteine residues 26 and 75, which clearly indicates that of the two neighboring cysteines (Cys74 and Cys75) in LILRA3 D1, Cys75 provides the correct conformation for native structure through its disulfide bond between the B and F strands.

LILRA3 D1 has a high level (>80%) of amino acid sequence identity with LILRB1 and B2. Figure 4C and 4D show the superimposed D1 structures between LILRA3 and LILRB1/B2. In these overlays, LILRA3 D1 is more similar to LILRB1 (RMSD = 0.348 for 1G0X, 0.336 for 1UFU) than LILRB2 (RMSD = 0.857 for 2GW5). There are some differences around the 78–83 loop region between LILRA3 D1 and LILRB1 D1 (Fig. 4C) and differences around the 29–33 and 77–83 loop regions between LILRA3 D1 and LILRB2 D1 (Fig. 4D). Though the loop regions display some differences between LILRA3 and LILRB1/B2, the amino acids in these regions do not participate in binding to HLAIs. Therefore, the differences in these regions may not affect the binding of LILRA3 to HLAIs.

### Structural basis for the interaction of LILRA3 with classical and non-classical HLAIs

**Figure 5:**
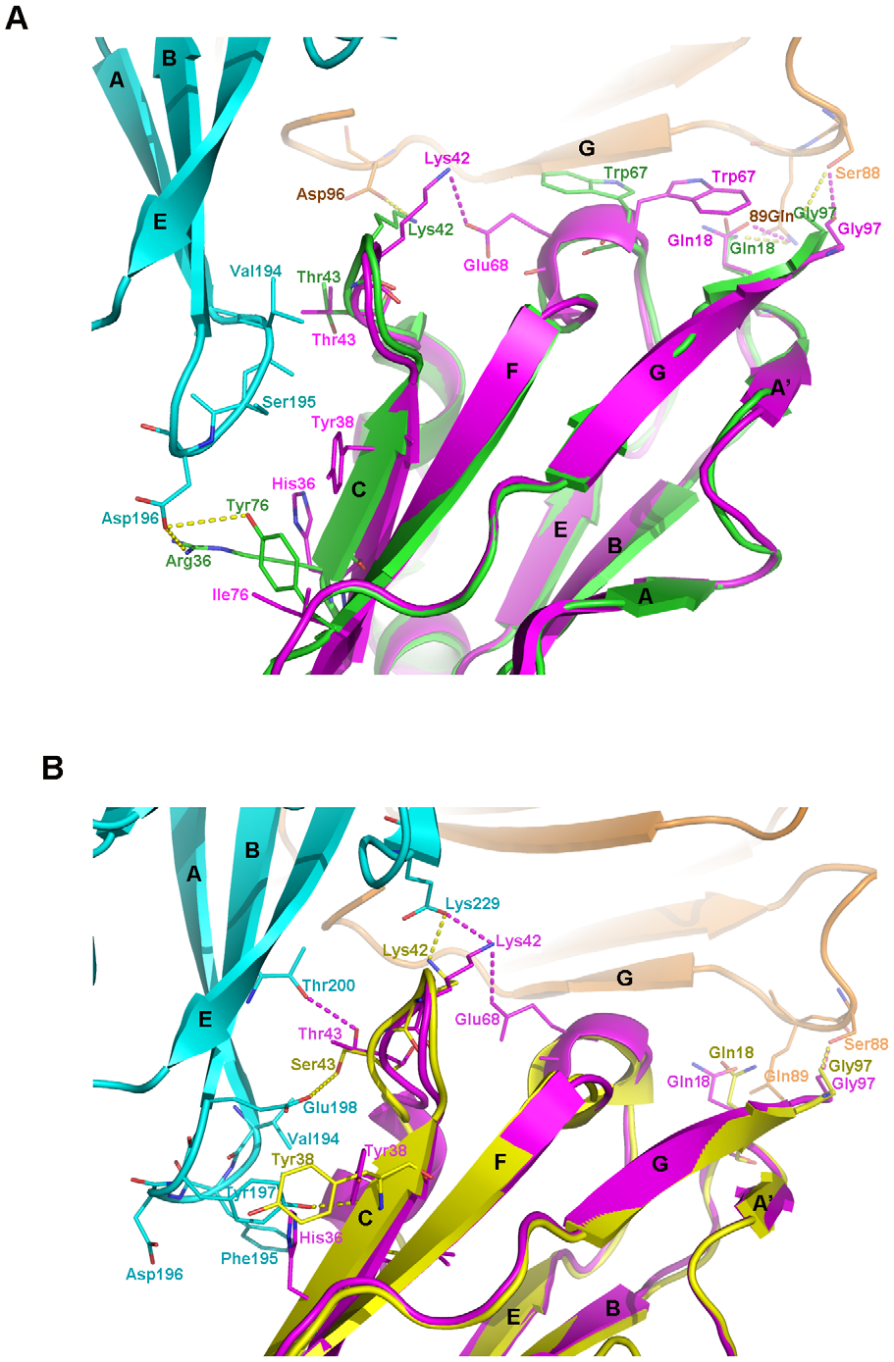
Comparison of the polar and hydrophobic interactions in the superposition of LILRA3 D1 (pink) to LILRB1/HLA-A*0201 (green) and LILRB2/HAL-G1 (yellow). A. The superposition of LILRA3 D1 to LILRB1/HLA-A*0201. Cyan and brown represent the α3 domain and the β2m of HLA-A*0201, respectively. The yellow dotted line represents the polar interaction between the residues of LILRB1 D1 and that of HLA-A*0201. The pink dotted line represents the polar interaction between the residues of LILRA3 D1 and that of HLA-A*0201. B. The superposition of LILRA3 D1 to LILRB2/HLA-G1. Cyan and brown represent the α3 domain and the β2m of HLA-G1, respectively. The yellow dotted line represents the polar interaction between the residues of LILRB2 D1 and that of HLA-G1. The pink dotted line represents the polar interaction between the residues of LILRA3 D1 and that of HLA-G1.

Previous studies using cytolysis or immuno-staining have not detected any significant binding of LILRA3 to HLAIs [13], [23], but our study using BIAcore® SPR demonstrated that LILRA3 binds both classical HLAI (HLA-A*0201) and non-classical HLAI (HLA-G1) with reduced binding affinity compared to LILRB1/HLA-A*0201 and LILRB2/HLA-G1 binding. Figure 5 shows the superposition of LILRA3 D1 with LILRB1/HLA-A*0201 (5A) and LILRB2/HLA-G1 (5B). Because the hydrophobic core composed of the C and E strands and the two 3_10_ helices between the strands provide the necessary conformation of the residues to interact with HLAIs, the important hydrophobic and polar interactions in these regions of LILRB1/HLA-A*0201 and LILRB2/HLA-G1 are conserved (Fig. 5A and 5B). LILRA3 Tyr38 forms hydrophobic interaction with the hydrophobic residues of the A-B loop of the α3 domain of HLA-A*0201 (Val194, Ser195, and Asp196) and the B strand of the α3 domain of HLA-G1 (Tyr197 and Glu198). Further, there may be a π-cation interaction between Lys91 of the β2m of HLA-A*0201 and Trp67 of LILRA3 D1, as seen with LILRB2 D1. The NH_3_^+^ of Lys91 in the β2m of HLA-A*0201 deeply intrudes into the aromatic ring of LILRA3 D1 Trp67, thereby strengthening the interaction between the two residues. As the distance between the Trp67 of LILRA3 D1 and the G strand of the HLA-A*0201 β2m becomes more intimate, Trp67 forms hydrophobic interactions with the hydrophobic residues (Ile92 and Val93) on the G strand of β2m. In the superimposed structure of LILRA3 and LILRB2/HLA-G1, the hydrophobic interactions of LILRB2 Ser43 with the residues (Val194 and Val248) of the α3 domain of HLA-G1 are also conserved as LILRA3 D1 Thr43 interacting with the same residues. Commonly existing interactions in both LILRB1/HLA-A*0201 and LILRB2/HLA-G1, such as the Gly97 (LILRB1/B2)-Ser88 (β2m of HLA-A*0201/G1) interaction (hydrogen bond) and Gln18 (LILRB1/B2)-Gln89 (β2m of HLA-A*0201/G1) interaction (hydrogen bond), are also conserved in the interaction between LILRA3 D1 and HLA-A*0201/G1. Thus, the crystal structure of LILRA3 D1 clearly indicates that LILRA3 would bind to both HLA-A*0201 and HLA-G1.

Meanwhile, structural explanations exist for the reduced binding affinity of LILRA3 for HLA-A*0201. Some polar interactions may not exist in the LILRA3 D1/HLA-A*0201 complex (Fig. 5A) because the amino acids at positions 36 and 76 were replaced with histidine and isoleucine from arginine (Arg36→His36) and tyrosine (Tyr76→Ile76). Thus, the salt bridge between Arg36, Tyr76 of LILRB1 and Asp196 of the α3 domain of HLA-A*0201 would be impossible in the LILRA3 D1/HLA-A*0201 interaction. Again, the crystal structure of LILRA3 D1 displays that Lys42 forms a hydrogen bond with Glu68 of LILRA3 D1, rendering Lys42 unable to bind to Asp96 of the α3 domain of HLA-A*0201. Indeed, the lack of polar interactions in the LILRA3 D1/HLA-A*0201 complex would reduce the binding affinity between these molecules. However, in the interaction between LILRA3 D1 and HLA-G1, most of the polar interactions are conserved (Fig. 5B). The superposition of LILRA3 D1 with LILRB2 D1D2/HLA-G1 shows a strong polar interaction with Tyr197 of the α3 domain of the heavy chain of HLA-G1. Both Leu37 and His36 in LILRA3 D1 form hydrogen bonds with Tyr197 of the α3 domain of the HLA-G1. Residue 43 of LILRA3 D1 is Thr, while in the LILRB2 D1, it is Ser, and there is a hydrogen bond between Ser43 of LILRB2 D1 and Glu198 of the HLA-G1 α3 domain. Because the hydroxyl of LILRA3 D1 Thr43 is oriented more closely toward Thr200 than Glu198 of the α3 domain of HLA-G1 (as compared to the hydrogen bond between Ser43 of LILRB2 D1 and Glu198 of the α3 domain of HLA-G1), Thr43 of LILRA3 D1 is able to form a hydrogen bond with Thr200 of the α3 domain of HLA-G1. LILRA3 Lys42 can also potentially form a hydrogen bond with Glu229 of the light chain of HLA-G1, as well as Glu68 of LILRA3 D1 itself. Thus, almost all polar interactions between HLA-G1 and LILRB2 D1 are conserved or compensated if LILRB2 D1 is replaced with LILRA3 D1. Indeed, the superposition of LILRA3 D1 with LILRB1/HLA-A*0201 and LILRB2/HLA-G1 clearly demonstrates that LILRA3 may binds to both classical and non-classical HLAIs and also explains well why LILRA3 would binds better to non-classical HLAIs than classical HLAIs. These conclusions are consistent with the SPR results.

### Key residues or structural elements crucial for influencing the binding affinity between Group 1 LILRs and HLAIs

As mentioned above, the changes of the amino acids at positions 36 and 76 between LILRA3 (His36 and Ile76) and LILRB1 (Arg36 and Tyr76) are one of the important reasons for the reduced binding affinity of LILRA3 to HLA-A*0201 compared to that of LILRB1 to HLA-A*0201. However, these changes do not affect the interaction between LILRA3 D1 and HLA-G1 because they favor the LILRB2/HLA-G1 binding mode. Leu37 in both LILRB2 and LILRA3 forms a hydrophobic interaction with Tyr197 of the α3 domain of HLA-G1, and Tyr76 does not participate in the interaction between LILRB2 and HLA-G1. Among Group 1 members, only LILRA3 and LILRA2 have a histidine at position 36. Other Group 1 members, including LILRA1, have Arg36 that fully interacts with Asp196 of HLA-A*0201. Therefore, we predict that LILRA1 will interact better with HLA-A*0201 than LILRA3.

Again, the self-interaction between LILRA3 D1 Lys42 and Glu68 is the other key structural difference that reduces the binding affinity of LILRA3 to HLA-A*0201 compared to that of LILRB1 to HLA-A*0201. The relatively rigid conformation of Lys42 due to the interaction with Glu68 makes it impossible for Lys42 to interact with Asp96 of the α3 domain of HLA-A*0201. However, in the LILRB2/HLA-G1 binding mode, Lys42 forms a hydrogen bond with Glu229 of the β2m of HLA-G1, as well as with Glu68 of LILRA3 D1.

**Figure 6:**
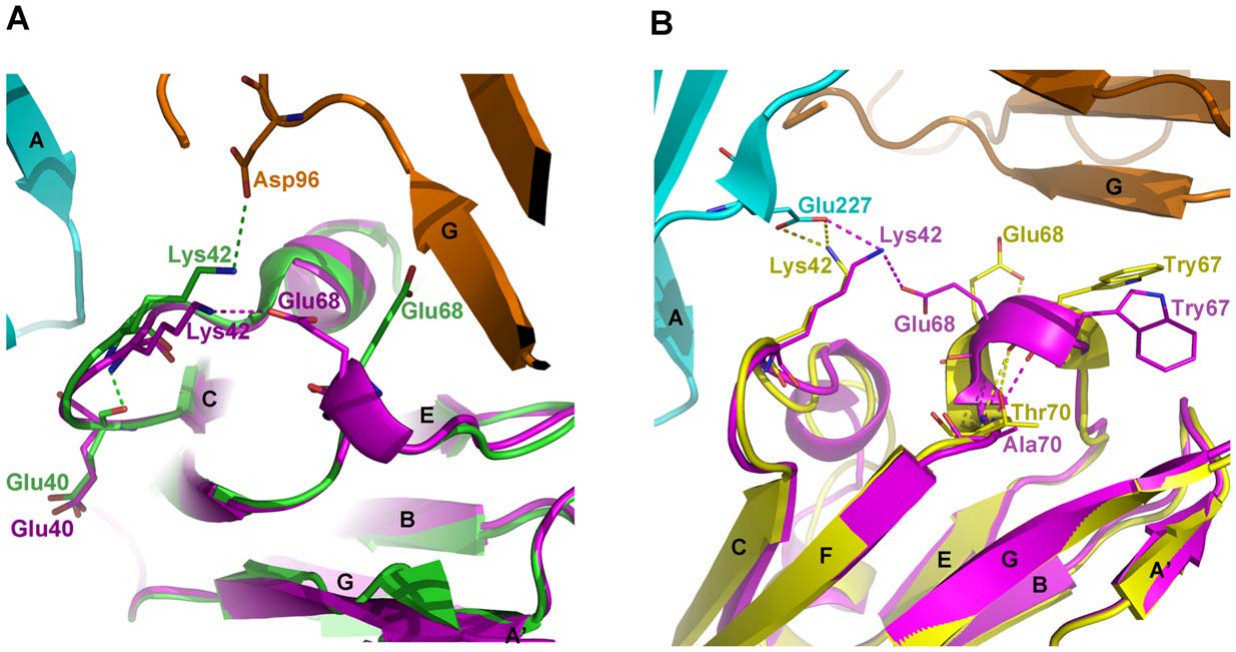
Comparison of the conformation of Lys42 of LILRB1 (green), LILRB2 (yellow), and LILRA3 (magenta) in complex with HLA-A*0201 or HLA-G1. A. Self-interaction of Lys42 with Glu68 in LILRA3 (pink dotted line) and the interaction of Lys42 of LILRB1 with Asp96 of the β2m of HLA-A*0201 in LILRB1/HLA-A*0201 (green dotted line). Cyan and brown represent the α3 domain and the β2m of HLA-A*0201, respectively. B. Self-interaction of Lys42 with Glu68 in LILRA3 (pink dotted line) and the interaction of Lys42 of LILRA3 (pink dotted line) and LILRB2 (yellow dotted line) with Glu227 of the α3 domain of HLA-G1. Cyan and brown represent the α3 domain and the β2m of HLA-G1, respectively.

Despite the high sequence similarity around Glu68 and Lys42 among LILRA3, LILRB1 and LILRB2, it is not clear why the Lys42 of LILRB1 and B2 do not form a self-interaction with Glu68. Figure 6 shows the detailed structure around Lys42 and Trp67 in the LILRA3 D1/LILRB1 D1 (Fig. 6A) and LILRA3 D1/LILRB2 D1 (Fig. 6B) structures. In all of the reported single crystal structures of LILRB1 D1D2 (1GOX, 1UFU, and 1UGN) and the co-crystal structure of LILRB1/HLA-A*0201, the peptide backbone carbonyl of LILRB1 D1 Glu40 forms a hydrogen bond with the nitrogen of the backbone amino group of Lys42, but in LILRA3 D1, there is no such interaction. Consequently, the loop of LILRA3 D1 between the C strand and 3_10_ helix 1 is relatively more flexible than that of LILRB1 D1, and the Lys42 on that loop may be able to form a hydrogen bond with Glu68 of the third 3_10_ helix of LILRA3 D1 (Fig. 6A). Contrary to this, LILRB1 D1 Lys42 is relatively rigid (due to its hydrogen bond with Glu40), and may be oriented only toward Asp96 of the β2m of HLA-A*0201. Meanwhile, in LILRB2 D1, there is no polar interaction between the backbones of Glu40 and Lys42, so the loop in question is flexible, as in LILRA3 D1. However, as shown in Figure 6B, there is also no hydrogen bond between Lys42 and Glu68 of LILRA3 D1. Additionally, the amino acids at position 70 are different between LILRA3 and LILRB2 (Ala and Thr, respectively), and the third 3_10_ helix structures are different, especially from the torsion angles between LILRA3 and LILRB2. Further, the orientations of Glu68 are quite different between LILRA3 and LILRB2; Glu68 of LILRB2 points toward the opposite direction of Lys42, making self-interaction impossible in LILRB2. Thus, though Group 1 LILRs display high amino acids conservation, one or two amino acid differences in the binding region or its adjacent region greatly influence the binding affinities for classical and non-classical HLAIs.

## Discussion

In this paper, we characterized the binding properties to HLAIs and D1 domain structure of LILRA3, an activating LILR Group 1 member. Our studies of LILRA3/HLAIs binding properties using BIAcore® SPR clearly demonstrated that LILRA3 binds both classical and non-classical HLAIs with reduced affinities compared to LILRB1/HLA-A*0201 and LILRB2/G1 binding. The crystal structure of LILRA3 D1 also supports these results. By comparing the binding region of LILRA3 D1 with LILRB1 D1 and LILRB2 D1 in the free structures or those complexed with HLAIs, the following conclusion can be drawn. First, as predicted, LILRA3 contains the fundamental fold for HLAI biding. Because it contains 3_10_ helices region located between the C and E strands of D1 and a hydrophobic core consisting of the helices and C and E strands, LILRA3 D1 can binds a broad range of classical and non-classical HLAIs. Second, one or two amino acid changes in the binding region of LILRA3 compared to LILRB1 and B2 reduce the binding affinity of LILRA3 to classical HLAIs relative to other Group 1 LILRs. Third, the self-interaction between Lys42 and Glu68 is unique to LILRA3 D1, and this property may be an important reason why the HLAI binding affinity of LILRA3 is reduced than that of LILRB1 and B2. In any event, LILRA1 follows this rule: if it displays self-interaction of Lys42 with Glu68, the binding affinity may be reduced relative to LILRB1 or B2. If not, the affinity will be greater than that of LILRA3. Indeed, on-going studies of the relationship between the binding affinity to HLAIs and the structure of LILRA1 will address this hypothesis.

LILRA3 is the only secretory LILR, and it is expressed by monocytes, macrophages, and dendritic cells, though mainly by macrophages [9], [24]. From a functional viewpoint, LILRA3 may greatly control the inhibition of the immune response induced by LILRB1, LILRB2, and other HLA-binding LILR molecules like LILRA1. However, why immune cells express different Group 1 LILRs with various binding affinities to a broad range of HLAIs and what is the important role of LILRA3 in macrophages must be addressed in the future. The crystal structure of the D1 domain and the SPR binding properties of LILRA3 indicated that LILRA3 binds HLA-G1 preferentially over HLA-A*0201, which explains its possible function in fetal-maternal immune control [34]. Indeed, β2m-free heavy chains (fHCs) of HLA-G1 are found in the placenta, and LILRA1 and LILRB2 can bind fHC tetramers of HLA-B27 and HLA-G1 [35]. The amino acid sequence alignment of Group 1 LILRs also predicts that LILRA3 recognizes classical HLAIs less well than LILRA1, LILRB1, and B2, as well as that LILRA3 may recognize the fHCs of classical HLAIs. This implies that LILRA3 would play an important role in the strict inhibitory control between classical and non-classical HLAIs. Interestingly, while in the revision process of this manuscript, Jones et al have just published the data showing that LILRA3 preferentially binds to HLA-C free heavy chain [36]. Thus, expression of LILRs with various affinities to different types of classical and non-classical HLAIs on a certain tissue or cell seems make the delicate organization of the immune response in the human body possible [2], [37]. Indeed, comparing the binding affinities between various LILR Group 1 members and multiple types of classical and non-classical HLAIs, as well as the possible role of LILRA3 in the inhibitory equilibrium, deserve considerable attention in the future.

## Materials and Methods

### Construction of the recombinant plasmids for LILRA3 D1 and D1D2

The full-length LILRA3 gene (containing D1–D4 and the stalk sequence) was cloned from a human lymphocyte cDNA library, as previously described [31], [38]. The primers used in the PCR amplification are as follows:

Forward primer = ACCCCCATCCTCACGGTCC, and
Reverse Primer = CTCACCAGCC TTGGAGTCGGA.

DNA fragments encoding the LILRA3 D1 and D1D2 regions (D1, 1–97; and D1D2, 1–197) were sub-cloned from the full-length gene, using the following primers:

D1/D1D2 Forward primer = GGAATTC*CATATG*GGGCCGCTCCCGAAGCCGACCC,
D1 Reverse Primer = CCG*CTCGAG*TTATCCTGTCACCACCAGCTCCAGGGGGT, and
D1D2 Reverse primer = CCG*CTCGAG*TTAACCTGGGACCAGGAGCCCCAGG.

In all forward and reverse primers, a start or stop codon was introduced with *Nde*I or *Xho*I restriction sites accordingly. All of the PCR products were ligated into the pET21a vector (Novagen), transformed into *E. coli* strain BL21 (DE3) pLysS, and expressed in the form of inclusion bodies.

### Protein preparation of LILRA3 D1, LILRA3 D1D2, LILRB2 D1D2, HLA-A*0201 and HLA-G1

Full-length LILRB2 DNA was kindly provided from Dr. Katsumi Maenaka, and the recombinant clone of its D1D2 region was prepared as previously reported [20]. The clones of the recombinant HLA-A*0201 and HLA-G1 were previously prepared [39].

All proteins were expressed in *E. coli* strain BL21 (DE3) pLysS and purified as previously reported [39], [40]. The inclusion body solution was injected into refolding buffer containing 100 mM Tris-HCl (pH 8.0), 2 mM EDTA, 400 mM L-arginine-HCl, 0.5 mM oxidized glutathione, 5 mM glutathione, 0.1 mM PMSF, and 0.1 mM NaN3. To purify the HLA-A2 and HLA-G1, the inclusion body solution of β2m and peptide (TLACFVLAAV, derived from human SARS-CoV membrane protein for HLA-A*0201; and RIIPRHLQL, derived from cellular endogenous protein histone for HLA-G1) dissolved in DMSO, were injected into the refolding buffer, and then the inclusion body solution of the heavy chain was later injected into the refolding buffer. The refolding solution was concentrated using a Stirred Cell and ultracentrifugal filter devices (Millipore). All proteins were purified by size exclusion chromatography using a Superdex 75 10/300 GL column (GE Healthcare), equilibrated in Tris-HCl (pH 8.0) buffer with an ÄCTA FPLC (Amersham Biosciences). For LILRA3 D1 and D1D2, the buffer was exchanged gradually to BIAcore buffer (HEPES, pH 7.4) and finally filtered on 200 10/300 GL column (GE Healthcare). For HLA-A*0201 and HLA-G1, the proteins were further purified by anion exchange chromatography (Resource Q) followed by final gel filtration on superdex 200 10/300 GL column (GE Healthcare) with BIAcore buffer (HEPES, pH 7.4). For ILT4 D1D2, it was also further purified by cation exchange chromatography (Source Q) followed by final gel filtration on superdex 200 10/300 GL column (GE Healthcare) with BIAcore buffer (HEPES, pH 7.4). The size exclusion chromatogram and the 12% SDS-PAGE of the main fractions confirmed the monomeric state of LILRB2 D1D2, HLA-A*0201, and HLA-G1.

### Crystallization of LILRA3 D1, data collection, phasing, and refinement

Despite efforts to crystallize LILRA3 D1D2, we failed to obtain any diffractive crystals. Therefore, we focused on crystal trials of LILR D1. The purified LILRA3 D1 was concentrated and adjusted to 15 mg/ml with elution buffer (50 mM NaCl, 20 mM Tris-HCl, pH 8.0), and crystallization was attempted at 18°C using the hanging drop vapor diffusion method. Crystallization conditions were optimized, and the best crystals were obtained using 1.0 M ammonium sulfate, 0.1 M HEPES buffer (pH 7.5), and 0.75% (w/v) PEG8000.

The LILRA3 D1 crystal was cryoprotected in mother liquor containing 15% glycerol before being flash-cooled in liquid nitrogen. Diffraction data were collected using an in-house X-ray source (Rigaku MicroMax007 desktop rotating-anode X-ray generator with a Cu target operated at 40 kV and 30 mA) and an R-AXIS IV++ imaging-plate detector at a wavelength of 1.5418 Å. The collected intensities were indexed, integrated, corrected for absorption, scaled, and merged using HKL-2000. Data collection and processing statistics are summarized in Table 1.

**Table 1:** X-ray diffraction data processing and refinement statistics.

Data collection	
Space group	C2
Cell dimensions	
*a*, *b*, *c* (Å)	83.31, 27.82, 48.26
α, β, γ (°)	90, 111.66, 90
Resolution (Å)	50-2.50(2.59-2.50)
*R*_sym_ or *R*_merge_	0.039(0.151)
*I*/σ	38.1(8.0)
Completeness (%)	96.7(92.5)
Redundancy	3.8(3.2)

Refinement	
Resolution (Å)	23.95-2.50
No. reflections	3503
*R*_work_/*R*_free_	0.2349/0.2619
No. atoms	
Protein	755
Ligand/ion	0
Water	32
*B*-factors	
Protein	44.9
Ligand/ion	0
Water	45.0
R.M.S. deviations	
Bond lengths (Å)	0.005
Bond angles (°)	1.025
Ramachandran plot	
Most favored (%)	87.5
Additionally favored (%)	11.3
Generally allowed (%)	1.3
Disallowed (%)	0

The structure of LILRA3 D1 was solved by the molecular replacement method using Phaser [41] from the CCP4 program suite [42] with the structure of LILRB2 (PDB code: 1VDG) as a search model. Initial restrained rigid-body refinement and manual model building were performed using REFMAC5 [43] and COOT [44], respectively. Further rounds of refinement were performed using the phenix.refine program, implemented in the PHENIX package [45], with coordinate refinement, isotropic ADP refinement, and bulk solvent modeling. The stereochemical quality of the final model was assessed with the PROCHECK program [46]. All of the presented structural figures were produced using PyMol (http://pymol.sourceforge.net).

### BIAcore® SPR analysis of LILRA3 D1 and D1D2

BIAcore®3000 was used to study the direct binding of LILRA3 D1 and D1D2 to HLA-A*0201 and HLA-G1. HLA-A*0201 and HLA-G1, as well as control protein (BSA), were individually immobilized (700, 300 and 700 resonance units (RU) respectively) by amine group coupling on research grade CM5 sensor chips at pH 5.0. LILRB2 D1D2, LILRA3 D1 and LILRA3D1D2 in HEPES buffer (10 mM HEPES (pH 7.4), 150 mM NaCl, 3 mM EDTA, and 0.005% (v/v) Tween-20) were then injected over the immobilized HLAIs at 25°C. Kinetic constants (*K*d) were derived by Scatchard analysis or nonlinear curve fitting of the standard Langmuir binding isotherm using BIAevaluation version 4.1 (BIAcore®).

### Protein Data Bank accession number

The atomic coordinates of LILRA3 D1 have been deposited in the RCSB Protein Data Bank with accession code of 3Q2C.
